# The logic of epistemic justification

**DOI:** 10.1007/s11229-017-1422-z

**Published:** 2017-05-24

**Authors:** Martin Smith

**Affiliations:** University of Edinburgh, Edinburgh, UK

**Keywords:** Justification, Probability, Normalcy, Risk minimisation theory, Normic theory

## Abstract

Theories of epistemic justification are commonly assessed by exploring their predictions about particular hypothetical cases—predictions as to whether justification is present or absent in this or that case. With a few exceptions, it is much less common for theories of epistemic justification to be assessed by exploring their predictions about *logical principles*. The exceptions are a handful of ‘closure’ principles, which have received a lot of attention, and which certain theories of justification are well known to invalidate. But these closure principles are only a small sample of the logical principles that we might consider. In this paper, I will outline four further logical principles that plausibly hold for justification and two which plausibly do not. While my primary aim is just to put these principles forward, I will use them to evaluate some different approaches to justification and (tentatively) conclude that a ‘normic’ theory of justification best captures its logic.

## Preliminaries: justification and conjunction

Consider the following principle for epistemic justification:

If one has justification for believing P and one has justification for believing Q, then one has justification for believing $\hbox {P}\,\wedge \,\hbox {Q}$.

This is sometimes called *conjunction closure*—if it is correct then the set of propositions that one has justification for believing, at any given time, is closed under the operation of taking conjunctions. The principle goes by other names too, such as *agglomeration* and *adjunction*. This can be described as a formal or *logical* principle, in that it features only a justification operator and logical constants. In fact, the principle could be written out as an inference schema in a modal logic, with a single monadic modal operator J, where J... is the proposition that one has justification for believing...:

$$\begin{aligned}&\hbox {JP}\\&\underline{\hbox {JQ}\qquad \,}\\&\hbox {J}(\hbox {P}\,\wedge \,\hbox {Q}) \end{aligned}$$

This inference will be valid in any so-called ‘normal’ modal logic^1^—though this, on its own, is no *argument* for accepting conjunction closure (I will detail a brief argument for it in the next section).

This principle has been the subject of considerable discussion amongst epistemologists, and features prominently in the lottery and preface paradoxes (see, for instance, Kyburg 1970, Pollock 1983, Foley 1993, chap. 4, Foley 2009, Douven And Williamson 2006). But aside from conjunction closure, and a few other exceptions, logical principles for justification feature little in contemporary epistemology^2^. And yet, logical principles can provide an invaluable resource for assessing theories of justification—an alternative to assessing such theories according to their predictions about hypothetical cases^3^. In this paper, I will present four further logical principles which justification might be thought to satisfy and two principles which it might be thought to violate. I will use these principles to assess the viability of certain approaches to justification, and conclude that, amongst these approaches, the logic of justification is best captured by a ‘normic’ theory. This paper will have met its primary aim, though, if this *method of assessment* be taken seriously—if these further logical principles be deemed worthy of serious consideration, alongside conjunction closure.

In the next section I will describe some of the philosophical background that led me to consider these logical principles, and also lay the groundwork for the conclusions that I will try to draw in the Sect. 4. None of this, though, is necessary to *state* the principles that I have in mind, or indeed to *evaluate* them. These principles are presented in Sect. 3, which effectively stands on its own.

## Prelimiaries: risk minimisation and its rivals

In previous work (Smith 2010, 2016) I’ve opposed a widespread view of epistemic justification that I’ve dubbed the ‘risk minimisation’ theory. According to this view, roughly speaking, one has justification for believing a proposition P just in case it would be unlikely, given one’s evidence, for P to be false. I’ve put forward an alternative that may seem very similar—almost like a terminological variant: One has justification for believing a proposition P just in case it would be *abnormal*, given one’s evidence, for P to be false. In one way, these theories *are* very close—they will make the same predictions about a broad range of hypothetical cases. In other ways, though, these theories are not at all alike—and focussing on the logic of justification is one way to bring out the differences between them.

Suppose I wander into a room I’ve never been in before and notice that the wall before me appears to be red. Given this evidence, it’s very likely that the wall before me *is* red. For a risk minimisation theorist there will have to be some probability threshold—0.9 or 0.95 or 0.99 etc.—at which a proposition is deemed ‘unlikely to be false’ and one enjoys justification for believing it. For any reasonable choice of threshold, we would want to say, in a case like this, that it would be unlikely for the proposition that the wall is red to be false. But would it also be *abnormal* for this proposition to be false?

To describe an event or a situation as ‘abnormal’ can, obviously, mean a number of different things. What we often intend to do, with such a description, is to mark out an event or situation as a *deviation* from a pattern or default—as something that would require special *explanation* if it were to occur or come about. If the wall appears to me to be red, but it *isn’t* red, then there would have to be some special explanation as to how this came to be—I’m undergoing a colour hallucination, the wall is illuminated by hidden red lights etc. It can’t *just so happen* that the wall appears to me to be red but isn’t—this is not the sort of thing that can ‘just so happen’.

In the case described, both the risk minimisation theory and the alternative ‘normic’ theory will predict that I have justification for believing that the wall is red—though they will offer slightly different accounts of why this is. Generally speaking, if the falsity of a proposition is unlikely, then the falsity of that proposition would also be abnormal, in the sense of calling for special explanation—and vice versa. At any rate, these properties do seem to go together in most of the examples that epistemologists have tended to focus upon. But they don’t *invariably* go together, as will soon become clear.

On the risk minimisation theory, there will be some probability threshold t, close to but less than 1, such that I have justification for believing all and only those propositions which, given my evidence, have probabilities above the threshold. On the risk minimisation conception, conjunction closure fails. This can be made vivid via the lottery and preface paradoxes, but the basic reason for the failure can be put simply: The probability of a conjunction can be lower than the probability of either conjunct and, as such, the probability of a conjunction may dip below the threshold for justification, even if the probability of each conjunct exceeds it. Suppose three friends—Jen, Bruce and Maude – have each told me that they’ll be attending the office Christmas party. Suppose I know Jen, Bruce and Maude to be very reliable and trustworthy and, as such, the propositions that Jen will attend, that Bruce will attend and that Maude will attend are each 95% likely, given my total evidence. Suppose finally that these propositions are mutually probabilistically independent of one another, so the probability of any one person attending is unaffected by whether or not any other person attends.

According to the risk minimisation theory, what do I have justification for believing? For ease, let’s set the threshold t at 0.9—so that I have justification for believing all and only those propositions that, given my evidence, are more than 90% likely to be true, and less than 10% likely to be false. In this case, I have justification for believing that Jen will attend, for believing that Bruce will attend and for believing that Maude will attend. I also have justification for believing that Jen and Bruce will attend, for believing that Jen and Maude will attend, and for believing that Bruce and Maude will attend—each of these propositions has a probability of $0.95^{2}=0.9025$. But I *lack* justification for believing that Jen and Bruce and Maude will all attend. This proposition has a probability of $0.95^{3}=0.857375$. These predictions are clearly at odds with conjunction closure.

If we pushed the threshold value t to 1, we would arrive at a kind of risk elimination or *infallibilist* theory of justification. On this theory, conjunction closure would be assured—if P has a probability of 1, given my evidence, and Q has a probability of 1, given my evidence, it follows that $\hbox {P}\,\wedge \,\hbox {Q}$ has a probability of 1 given my evidence. But infallibilism carries a steep price—making justification very difficult, or even impossible, to attain^4^. And, in the present example, conjunction closure would only end up being satisfied in a trivial way—on the infallibilist theory, I wouldn’t have justification for believing that Jen will attend or for believing that Bruce will attend or for believing that Maude will attend, or for believing any substantial proposition about who will be attending the party.

What about the normic theory? Given Jen’s reliability and her commitment to attend the party, it would be abnormal if she failed to show up, and some explanation would be needed—illness, car trouble, a family emergency etc. The same goes for Bruce and for Maude. The normic theory predicts that I have justification for believing that Jen will attend, for believing that Bruce will attend and for believing that Maude will attend. But it also predicts that I have justification for believing the conjunction that Jen and Bruce and Maude will all attend. After all, if this proposition is false then at least one of the three must fail to attend the party, in spite of committing to it, and this would require some special explanation—illness, car trouble, family emergency etc. The normic theory is not an infallibilist theory, yet its predictions are consistent with conjunction closure—at least in this case.

Conjunction closure is a special instance of the principle of *deductive closure* according to which, if one has justification for believing each of a series of propositions $\hbox {P}_{1}$, $\hbox {P}_{2}$...$\hbox {P}_{\mathrm{n}}$ and $\hbox {P}_{1}$, $\hbox {P}_{2}$...$\hbox {P}_{\mathrm{n}}$ deductively entail Q, then one has justification for believing Q. Most philosophers would agree that these closure principles do have *some* intuitive appeal. It is natural to draw deductive inferences using the propositions that we believe – and unsettling to think that, by doing this, we could be led from propositions that are justified to propositions for which we lack justification. Beyond this observation, though, philosophers tend to take very different attitudes towards these principles. Some philosophers appear to regard deductive closure and conjunction closure as almost *sacrosanct*—constraints that any adequate theory of justification must satisfy. For philosophers coming from this starting point, the example just described would constitute a powerful objection to the risk minimisation theory. For others, though, it’s wrong to trust our gut reaction to these principles – we can’t make an informed judgment about them until we have an adequate *theory* of epistemic justification to guide us. For a risk minimisation theorist coming from this perspective, the failure of conjunction closure is simply a *result*, rather than an objection to the theory. I won’t attempt to pursue this dispute any further here (I’m unsure how to pursue it further). What I do think, though, is that conjunction closure is not the only principle at stake in this example.

Many epistemologists have been attracted to some version of the following idea: If one has justification for believing a proposition P, then one also has justification for using P as a premise in one’s practical and theoretical reasoning (see for instance Fantl and McGrath 2011, chapter 4). Let’s put practical reasoning to one side, and focus just on the theoretical; the idea, then, is that whenever one has justification for believing a proposition, one can justifiably treat it as another piece of evidence from which to draw further conclusions and with which to justify further beliefs. Most who put forward this claim would, I think, regard it as something that precedes substantial theorising about justification—as something that helps to delimit the very notion that we’re theorising about.

What does this have to do with the present example? According to the risk minimisation theory, as we’ve seen, I have justification for believing that Jen will attend, but I lack justification for believing that Jen and Bruce and Maude will attend. But if I could simply *add* the proposition that Jen will attend to my stock of evidence, then I *would* have justification for believing that Jen and Bruce and Maude will attend—with the independence assumption in place, the probability that Jen and Bruce and Maude will attend, given my evidence *and* the proposition that Jen will attend, is 0.9025, which exceeds the threshold. Consider the following:

If one has justification for believing P and one’s evidence, along with P, provides justification for believing Q, then one has justification for believing Q.

This principle has a good claim to regimenting the idea that, when we have justification for believing a proposition, we also have justification for using it as a premise in our theoretical reasoning. This principle guarantees that, if I have justification for believing a proposition, and I treat that proposition as another piece of evidence in the service of justifying further beliefs, I won’t be led to believe any propositions for which I lack justification. This is another logical principle for justification (though not one that can be written out in a monadic modal logic).

If we accept the risk minimisation theory then we have to give up not only conjunction closure, but this logical principle as well. My original interest in this principle owed to the fact that it clashed with the risk minimisation theory. Rather than weighing into the deadlocked dispute over conjunction closure, it seemed to me that the principle could be used to ‘outflank’ the proponents of risk minimisation—and attack the theory from another side. Whatever one thinks about the prospects for this, the present considerations naturally arouse our curiosity as to whether there are any other logical principles against which the risk minimisation theory, and other theories of justification, could be tested. In the next section I will explore a number of further logical principles for justification. I will return to the risk minimisation and normic theories in the final section.

## The logic of epistemic justification

Two assumptions will significantly expand the range of logical principles for justification that we can write down. The first of these is the ‘evidentialist’ assumption that justification is provided by evidence—if one has justification for believing a proposition then this justification is provided by the total evidence that one possesses. The second is the ‘propositionalist’ assumption that one’s evidence consists of a stock of propositions, or a conjunction of propositions. With both assumptions in place, the justificatory relation between a subject and a proposition can be analysed in terms of a relation *between propositions* – what it is for one to have justification for believing a proposition is for the propositions in one’s evidence to provide justification for believing it. In this case, justification is best captured not by a monadic modal operator, but a *dyadic* one—a *conditional*, in effect.

I won’t discuss the evidentialist and propositionalist assumptions at length. Suffice it to say though, in order for these assumptions to play their present role, they need only be taken in a very minimal way. Some epistemologists have attempted to drive a wedge between justification and evidence, arguing that certain non-evidential factors can play a role in providing justification (see for instance Bergmann 2006, chap. 5). Views of this kind tend, however, to be predicated upon substantial assumptions about what evidence is—often the assumption that our evidence must be something to which we have special or privileged access. When the notion of evidence is freed from these associations, these views need not be at odds with the evidentialist assumption. Indeed, there is a broad sense of ‘evidence’ on which one’s total evidence can be taken to include whatever facts about one’s epistemic position are taken to bear upon one’s overall justificatory status.

Some epistemologists have also denied that evidence is propositional, arguing that one’s evidence consists instead of one’s experiences and relevant mental states (see for instance Conee and Feldman 2008, for some discussion see Williamson 2000, section 9.5, Dougherty 2011). But this position is compatible with the term ‘evidence’ being extended, at least in a derivative sense, to the propositions describing one’s experiences and relevant mental states. If one’s justificatory status is determined by the former, it will also be determined by the latter.

In the previous section, I set out the following principle: If one has justification for believing P and one’s evidence, along with P, provides justification for believing Q then one has justification for believing Q. Given the evidentialist and propositionalist assumptions, the three components of the principle can be analysed as having the same logical form. If we let E be the conjunction of propositions in one’s evidence, the principle becomes: If E provides justification for believing P and $\hbox {E}\,\wedge \,\hbox {P}$ provides justification for believing Q then E provides justification for believing Q^5^. This could be written out as an inference schema in a conditional logic with a single conditional operator $=>$ where __ $=>$... is the proposition that __ provides justification for believing...:

$$\begin{aligned}&\hbox {E}=>\hbox {P}\\&(\underline{\hbox {E}\,\wedge \,\hbox {P}) =>\hbox {Q}}\\&\hbox {E} => \hbox {Q} \end{aligned}$$

We might call this principle *cumulative transitivity* (a name that only makes sense once we see the principle written out in this way).

In fact, the conjunction closure principle also corresponds to an inference schema in this logic, where E is one’s body of evidence:

$$\begin{aligned}&\hbox {E}=>\hbox {P}\\&\underline{\hbox {E} =>\hbox {Q}\qquad }\\&\hbox {E} => (\hbox {P}\,\wedge \,\hbox {Q}) \end{aligned}$$

The reason this inference can be captured in a simpler formalism is that there is no variation in the antecedent term, enabling us, in effect, to condense $\hbox {E} =>$ into a monadic operator^6^. Return, though, to cumulative transitivity. A simple transitivity pattern for $=>$ would be as follows:

$$\begin{aligned}&\hbox {E}=>\hbox {P}\\&\underline{\hbox {P}=>\hbox {Q}}\\&\hbox {E}=>\hbox {Q} \end{aligned}$$

But this seems *not* to be valid, given the intended interpretation of ‘$=>$’.

Let L be the proposition that the wall is white and illuminated by tricky red light, A be the proposition that the wall appears to be red and R be the proposition that the wall is red. Plausibly, the proposition that the wall is white and illuminated by tricky red light provides justification for believing that the wall appears to be red, and the proposition that the wall appears to be red provides justification for believing that the wall is red. But the proposition that the wall is white and illuminated by tricky red light does *not* provide justification for believing that the wall is red. We have $\hbox {L} => \hbox {A}$ and $\hbox {A} => \hbox {R}$ and $\hbox {L} \ne > \hbox {R}$. This is not a counterexample to cumulative transitivity however. The proposition that the wall appears to be red *and* is white and illuminated by tricky red light does not provide justification for believing that the wall is red. We have $(\hbox {L} \,\wedge \, \hbox {A})\,\ne > \hbox {R}$.

The intuitive motivation for cumulative transitivity was noted in the last section: It’s very plausible to think that, if I have justification for believing a proposition P, then I also have justification for using that proposition as a premise in theoretical reasoning. But what does it mean to be justified in using a proposition in theoretical reasoning? Part of what this means is that, by using the proposition to justify further beliefs, I won’t be led to believe any propositions for which I lack justification. Naturally, though, the proposition must be used within the in the context of my *total* evidence, including the evidence that provided my justification for believing it. If I were to ignore my existing evidence, then I might well be led to believe propositions for which I lack justification. It is for this reason that simple transitivity fails for $=>$.

Simple transitivity could be derived from cumulative transitivity, if we were able make use of the following schema, sometimes termed *monotonicity*:

$$\begin{aligned}&\underline{\hbox {E} => \hbox {P}\qquad }\\&(\hbox {E}\,\wedge \,\hbox {Q}) => \hbox {P} \end{aligned}$$

If we had the premises $\hbox {L} => \hbox {A}$ and $\hbox {A} => \hbox {R}$ then, by monotonicity, we would have $(\hbox {L} \,\wedge \, \hbox {A}) =>\hbox {R}$ and, by cumulative transitivity, we would have $\hbox {L} => \hbox {R}$ as required^7^. Monotonicity is clearly not a valid schema, with its invalidity reflecting the fact that justification is *defeasible*. Just because a certain body of evidence provides justification for believing a proposition, it doesn’t follow that an enriched or augmented body of evidence will continue to do so. Just because I possess evidence that provides justification for believing a proposition, it doesn’t follow that my *total* evidence provides justification for believing it.

The failure of monotonicity, for a logical consequence relation, has been the subject of extensive investigation in nonmonotonic logic, where a number of weakenings of the monotonicity property have been described. Two of these weakenings, sometimes termed *cautious monotonicity* and *rational monotonicity* can each be arrived at by adding a premise to the monotonicity schema (see for instance Lehman and Magidor 1992, sections 2 and 3, Hawthorne 1996, section 3, 2007, sections 3 and 4). The cautious monotonicity schema looks like this:

$$\begin{aligned}&\hbox {E} => \hbox {P}\\&\underline{\hbox {E} => \hbox {Q}\qquad }\\&(\hbox {E}\,\wedge \, \hbox {Q}) => \hbox {P} \end{aligned}$$

And the rational monotonicity schema is this:

$$\begin{aligned}&\hbox {E} => \hbox {P}\\&\underline{\hbox {E} \ne>{\sim }\hbox {Q}\qquad }\\&(\hbox {E} \,\wedge \, \hbox {Q}) => \hbox {P} \end{aligned}$$

Are these schemas valid, given the intended interpretation of ‘$=>$’? If I justifiably believe a proposition, and my justification is vulnerable to a certain defeater then, in so far as I consider the issue, I would—and should—believe that the defeater does not obtain. To be agnostic about the defeater, while clinging on to my belief, would seem incoherent. But, in order to ensure that I would be justified in believing that the defeater does not obtain, we would need something like the following principle:

If I have justification for believing P, and Q defeats my justification for believing P, then I have justification for believing that Q is false.

Rational monotonicity is nothing more than this principle, written out as an inference schema. The claim that I have justification for believing P becomes $\hbox {E} => \hbox {P}$, the claim that Q defeats my justification for believing P becomes $(\hbox {E} \,\wedge \, \hbox {Q}) \ne > \hbox {P}$, and the claim that I have justification for believing that Q is false becomes $\hbox {E}~={>}{\sim }\hbox {Q}$. We have it that $\hbox {E}~={>}{\sim }\hbox {Q}$ follows from $\hbox {E} => \hbox {P}$ and $(\hbox {E} \,\wedge \, \hbox {Q}) \ne > \hbox {P}$ which is just to say that $(\hbox {E} \,\wedge \, \hbox {Q}) => \hbox {P}$ follows from $\hbox {E} => \hbox {P}$ and $\hbox {E} \ne {>}{\sim }\hbox {Q}$^8^.

Here is an even more secure principle linking justification and defeat:

If I have justification for believing P and Q defeats my justification for believing P, then I don’t also have justification for believing that Q is true.

Cautious monotonicity is nothing more than this principle written out as an inference schema. The claim that I have justification for believing P becomes $\hbox {E} => \hbox {P}$, the claim that Q defeats my justification for believing P becomes $(\hbox {E} \,\wedge \, \hbox {Q}) \ne > \hbox {P}$ and the claim that I don’t have justification for believing that Q is true becomes $\hbox {E} \ne > \hbox {Q}$. We have it that $\hbox {E} \ne > \hbox {Q}$ follows from $\hbox {E} => \hbox {P}$ and $(\hbox {E} \,\wedge \, \hbox {Q})\,\ne > \hbox {P}$ which is just to say that $(\hbox {E} \,\wedge \, \hbox {Q}) => \hbox {P}$ follows from $\hbox {E} => \hbox {P}$ and $\hbox {E} => \hbox {Q}$.

Finally, consider the following: If I know that a given investigation is bound to yield justification for believing P, then I already have justification for believing P. We might call this the ‘no need’ principle. If I’m only interested in P, and I know that an investigation will provide justification for believing P, however it turns out, then it seems there’s *no need* to go ahead with the investigation. I’m unsure whether this idea can be fully captured with a logical principle, but I do think that a certain logical principle can be extracted from it. One way we might know that an investigation is bound to provide justification for believing P is if it has only two possible outcomes—either it will yield proposition E or proposition F as evidence—and each of these propositions would provide justification for believing P. If these are really the only possible outcomes of the investigation then, without conducting the investigation, I already have the evidence $\hbox {E} \,\vee \, \hbox {F}$. According to the no need principle this should already be enough to provide justification for believing P. This gives us the following formal principle:

If E provides justification for believing P and F provides justification for believing P then $\hbox {E} \,\vee \, \hbox {F}$ provides justification for believing P.

This can clearly be written out as an inference schema, sometimes called *amalgamation*:

$$\begin{aligned}&\hbox {E} => \hbox {P}\\&\underline{\hbox {F} => \hbox {P}\qquad }\\&(\hbox {E} \,\vee \, \hbox {F}) => \hbox {P} \end{aligned}$$

If amalgamation is correct, then the set of propositions that can justify a given proposition is closed under the operation of taking disjunctions. In a way, amalgamation is the flipside of the agglomeration or conjunction closure principle. If we hold the antecedent of a set of justificatory conditionals constant, agglomeration allows us to freely conjoin their consequents. If we hold the consequent of a set of justificatory conditionals constant, amalgamation allows us to freely disjoin their antecedents.

## Risk minimisation again

On the risk minimisation theory, there will be a probability function Pr such that $\hbox {E} => \hbox {P iff Pr}(\hbox {P} \,|\, \hbox {E}) > \hbox {t}$, for some t close to but less than 1^9^. On the risk minimisation theory, the $=>$ operator will become what Hawthorne (1996) terms a ‘probability-like’ conditional. In this case, the logical properties of $=>$ will be dictated by the logical properties of Pr. A probability function is nothing more than a function mapping propositions to numbers in a way that meets certain constraints. The domain of a probability function is a set of propositions F that is closed under negation and disjunction and includes a maximal proposition entailed by all others in the set. Propositions are often modelled, for this purpose, as subsets of a set of possible worlds W, with W itself serving as the maximal proposition. A (classical) probability function Pr takes each proposition in the set to a real number in a way that conforms to the following axioms:

1. $$\hbox {Pr}(\hbox {W}) = 1$$
2. $$\hbox {Pr}(\hbox {P}) \ge 0$$
3. If P and Q are inconsistent then $\hbox {Pr}(\hbox {P} \,\vee \, \hbox {Q}) = \hbox {Pr}(\hbox {P}) + \hbox {Pr}(\hbox {Q})$

Conditional probability is generally defined by the ratio formula: $\hbox {Pr}(\hbox {P} \,|\, \hbox {E}) = \hbox {Pr}(\hbox {P} \,\wedge \, \hbox {E})/\hbox {Pr}(\hbox {E})\hbox { if Pr}(\hbox {E})>0$ and is undefined otherwise^10^. P and Q are said to be independent just in case $\hbox {Pr}(\hbox {P} \,|\, \hbox {Q}) = \hbox {Pr}(\hbox {P})$ which, given the ratio formula, entails that $\hbox {Pr}(\hbox {P} \,\wedge \, \hbox {Q}) = \hbox {Pr}(\hbox {P}).\hbox {Pr}(\hbox {Q})$. Further, P and Q are said to be independent, given E, just in case $\hbox {Pr}(\hbox {P} \,|\, \hbox {Q} \,\wedge \, \hbox {E}) = \hbox {Pr}(\hbox {P} \,|\, \hbox {E})$ which, given the ratio formula, entails that $\hbox {Pr}(\hbox {P} \,\wedge \, \hbox {Q} \,\,|\, \hbox {E}) = \hbox {Pr}(\hbox {P} \,|\, \hbox {E}).\hbox {Pr}(\hbox {Q} \,\,|\, \hbox {E})$. The following complementation principle is an obvious consequence of (P1) and (P3):

(P4) $$\hbox {Pr}({\sim }\hbox {P}) = 1 - \hbox {Pr}(\hbox {P})$$

Given the definition of conditional probability, it also has a conditional version:

(P5) If $$\hbox {Pr}(\hbox {Q}) > 0$$ then $$\hbox {Pr}(\hbox {P} \,|\, \hbox {Q}) = 1 - \hbox {Pr}({\sim }\hbox {P} \,|\, \hbox {Q})$$

We have already seen that the risk minimisation theory invalidates conjunction closure and cumulative transitivity—this was illustrated in Sect. 2 using a simple example. In fact, *every one* of the formal principles listed in the last section will fail on the risk minimisation theory—and can be shown to fail in this same simple example. In the example Jen, Bruce and Maude each committed to attending the office Christmas party making the attendance of each (so it was stipulated) 95% likely. Let T be the proposition that Jen, Bruce and Maude have testified that they will attend, J be the proposition that Jen will attend, B be the proposition that Bruce will attend and M be the proposition that Maude will attend, and let Pr be my evidential probability function, prior to the receipt of the testimony. We have it that $\hbox {Pr}(\hbox {J} \,|\, \hbox {T}) = 0.95$, $\hbox {Pr}(\hbox {B} \,|\, \hbox {T}) = 0.95$ and $\hbox {Pr}(\hbox {M} \,\,|\, \hbox {T}) = 0.95$. It was also stipulated that J, B and M are mutually independent and mutually independent given T, in which case $\hbox {Pr}(\hbox {J} \,\wedge \, \hbox {B} \,\wedge \, \hbox {M} \,\,|\, \hbox {T}) = 0.95^{3} = 0.857375$ and $\hbox {Pr}({\sim }\hbox {J} \,\wedge \,{\sim }\hbox {B} \,\wedge \,{\sim }\hbox {M} \,\,|\, \hbox {T}) = 0.05^{3} = 0.000125$. By (P5), $\hbox {Pr}({\sim }\hbox {J}\,\vee \,{\sim }\hbox {B} \,\vee \,{\sim }\hbox {M}) \,|\, \hbox {T}) = 1 - 0.857375 = 0.142625$ and $\hbox {Pr}(\hbox {J} \,\vee \, \hbox {B} \,\vee \, \hbox {M} \,\,|\, \hbox {T}) = 1 - 0.000125 = 0.999875$.

From this, it can be calculated that $\hbox {Pr}(\hbox {J}~|~\hbox {T}~\,\wedge \, ~({\sim }\hbox {J} \,\vee \,{\sim }\hbox {B} \,\vee \,{\sim }\hbox {M})) \approx 0.649$ (the details are left to the reader). Suppose we set the threshold value t at 0.9 – so that $\hbox {E} => \hbox {P iff Pr}(\hbox {P} \,|\, \hbox {E}) > 0.9$. In this case we have it that $\hbox {T} => \hbox {J}, (\hbox {T}~\,\wedge ~({\sim }\hbox {J} \,\vee \,{\sim }\hbox {B} \,\vee {\sim }\hbox {M})) \ne > \hbox {J}$ and $\hbox {T}\ne > (\hbox {J} \,\wedge \, \hbox {B} \,\wedge \, \hbox {M})$. While I have justification for believing that Jen will attend, vulnerable to defeat by the proposition that one of the three will fail to attend, I lack justification for believing that this proposition is false. The predictions of the risk minimisation theory are inconsistent with rational monotonicity.

When it comes to cautious monotonicity, the counterexample is a little more difficult to extract. By independence $\hbox {Pr}(\hbox {J} \,\wedge \, \hbox {B} \,|\, \hbox {T}) = \hbox {Pr}(\hbox {J} \,|\, \hbox {T}).\hbox {Pr}(\hbox {B} \,|\, \hbox {T}) = 0.9025$ and, by (P5), $\hbox {Pr}(\hbox {M}~\,\vee \, ~{\sim }\hbox {J}~\,\vee \, ~{\sim }\hbox {B}~| \hbox {T}) = 1 - \hbox {Pr}({\sim }\hbox {M} \,\wedge \, \hbox {J}\,\wedge \, \hbox {B}\,|\, \hbox {T}) = 1 - (0.95^{2} \times 0.05) = 0.954875$. We can then calculate that $\hbox {Pr}(\hbox {J}~\,\wedge \, ~\hbox {B}~|~(\hbox {M}~\,\vee \, ~{\sim }\hbox {J}~\,\vee \,{\sim }\hbox {B})~\,\wedge \, \hbox {T})\approx 0.898$ (the details once again left to the reader). We have it that $\hbox {T} => (\hbox {J}~\,\wedge \, \hbox {B})$, $\hbox {T} => (\hbox {M} \,\vee \, {\sim }\hbox {J}\,\vee \,{\sim }\hbox {B})$ and $(\hbox {T}\,\wedge \, (\hbox {M} \,\vee \, {\sim }\hbox {J} \,\vee \,{\sim }\hbox {B})) \ne > (\hbox {J}\,\wedge \, \hbox {B})$. In this case I have justification for believing that Jen and Bruce will attend, vulnerable to defeat by the proposition that either Maude will attend or Jen or Bruce will fail to. But I also have justification for believing that either Maude will attend or Jen or Bruce will fail to. The predictions of the risk minimisation theory are inconsistent with cautious monotonicity.

Finally, notice that, given independence and the definition of conditional probability, $\hbox {Pr}(\hbox {J}~\,\wedge \, ~\hbox {B}~\,\wedge \, \hbox {M} \,\,|\, \hbox {J} \,\wedge \, \hbox {T}) = \hbox {Pr}(\hbox {B} \,\wedge \, \hbox {M} \,\,|\, \hbox {T}) = 0.9025$. Similarly, $\hbox {Pr}(\hbox {J}~\,\wedge \, ~\hbox {B}~\,\wedge \, ~\hbox {M}~|~\hbox {B}~\,\wedge \, ~\hbox {T}) = 0.9025$ and $\hbox {Pr}(\hbox {J}~\,\wedge \, ~\hbox {B}~\,\wedge \, ~\hbox {M}~|~\hbox {M}~\,\wedge \, ~\hbox {T}) = 0.9025$. But it is then possible to calculate that $\hbox {Pr}(\hbox {J} \,\wedge \, \hbox {B} \,\wedge \,\, \hbox {M} \,|\, (\hbox {J} \,\vee \, \hbox {B} \,\vee \, \hbox {M}) \,\wedge \, \hbox {T}) \approx 0.8574$. We have it that $(\hbox {J} \,\wedge \, \hbox {T}) => (\hbox {J} \,\wedge \, \hbox {B} \,\wedge \, \hbox {M})$, $(\hbox {B} \,\wedge \, \hbox {T}) => (\hbox {J} \,\wedge \, \hbox {B}\,\wedge \, \hbox {M})$ and $(\hbox {M}\,\wedge \, \hbox {T}) => (\hbox {J} \,\wedge \, \hbox {B} \,\wedge \, \hbox {M})$ but $((\hbox {J}~\,\vee \, ~\hbox {B}~\,\vee \, ~\hbox {M}) \,\wedge \, \hbox {T}) \ne > (\hbox {J} \,\wedge \, \hbox {B}\,\wedge \, \hbox {M})$. If I have the evidence that Jen will attend, I have justification for believing that all three will attend. If I have the evidence that Bruce will attend, I have justification for believing that that all three will attend. If I have the evidence that Maude will attend, I have justification for believing that that all three will attend. But if I have the evidence that Jen *or* Bruce *or* Maude will attend, I don’t have justification for believing that that all three will attend. The predictions of the risk minimisation theory are inconsistent with amalgamation.

We could easily devise more straightforward counterexamples to these principles, specially tailored to each one. Sticking with this one simple example highlights, though, just how widespread the failure of these principles will be on the risk minimisation conception. It’s not just that these principles will break down in this or that special circumstance—they will break down, in some way, in almost any case we can imagine.

There is a straightforward modification we can make to the risk minimisation theory that will lead to an enormous formal difference. As noted in Sect. 2, if we push the threshold value t to all the way to 1 then conjunction closure will be valid. The same goes for cumulative transitivity, cautious and rational monotonicity and amalgamation. If $\hbox {Pr}(\hbox {P} \,|\, \hbox {E}) = 1$ and $\hbox {Pr}(\hbox {Q} \,\,|\, \hbox {E} \,\wedge \, \hbox {P}) = 1$, it follows that $\hbox {Pr}(\hbox {Q} \,\,|\, \hbox {E}) = 1$. If $\hbox {Pr}(\hbox {P} \,|\, \hbox {E}) = 1$ and $\hbox {Pr}(\hbox {Q} \,\,|\, \hbox {E}) = 1$ it follows that $\hbox {Pr}(\hbox {P} \,|\, \hbox {E} \,\wedge \, \hbox {Q}) = 1$. Furthermore, this inference goes through even if the second premise is replaced with the weaker $\hbox {Pr}({\sim }\hbox {Q} \,\,|\, \hbox {E}) < 1$. Finally, if $\hbox {Pr}(\hbox {P} \,|\, \hbox {E}) = 1$ and $\hbox {Pr}(\hbox {P} \,|\, \hbox {F}) = 1$, it follows that $\hbox {Pr}(\hbox {P} \,|\, \hbox {E} \,\vee \, \hbox {F}) = 1$.

Most, though, would consider this kind of infallibilist view to be a complete nonstarter—so many of the things we take ourselves to have justification for believing are not made certain by our evidence (modulo the possibilities mentioned in n4). While the infallibilist theory may secure the logical properties for justification that we desire, it does so at the cost of mishandling almost every particular case. Perhaps there is a sobering lesson here about using logical principles to decide on a theory of justification—perhaps we should always let particular cases serve as the ultimate arbiter. This thought is too quick though—for even if we do just restrict attention to logic, infallibilism has a far from unblemished record. While it does validate conjunction closure, cumulative transitivity, cautious and rational monotonicity and amalgamation, infallibilism will also serve to validate *simple* transitivity^11^. If $\hbox {Pr}(\hbox {P} \,|\, \hbox {E}) = 1$ and $\hbox {Pr}(\hbox {Q} \,\,|\, \hbox {P}) = 1$, it follows that $\hbox {Pr}(\hbox {Q} \,\,|\, \hbox {E}) = 1$. The infallibilist theory will also, in a sense, validate monotonicity—if $\hbox {Pr}(\hbox {P} \,|\, \hbox {E}) = 1$ it follows that $\hbox {Pr}(\hbox {P}~|~\hbox {E}~\,\wedge \, ~\hbox {Q}) = 1$ if $\hbox {Pr}(\hbox {P}~|~\hbox {E}~\,\wedge \, ~\hbox {Q})$ is defined^12^. While the risk minimisation theory falls short, leaving justification with too little logical structure, infallibilism overshoots the mark, saddling justification with *too much*.

What, then, of the normic theory? According to the normic theory, I have justification for believing a proposition P just in case, given my evidence, the falsity of P would be *abnormal* in the sense of calling for special explanation. For some of the logical principles I’ve considered, it’s relatively easy to see why this theory should validate them. Consider conjunction closure. Suppose my evidence E provides justification for believing P and justification for believing Q. According to the normic theory, there would have to be some special explanation if E were true and P were false and there would have to be some special explanation if E were true and Q were false. What about $\hbox {P}~\,\wedge \, ~\hbox {Q}$? If $\hbox {P} \,\wedge \, \hbox {Q}$ were false then *either* P would be false *or* Q would be false. As such, there would have to be some special explanation if E were true and $\hbox {P} \,\wedge \, \hbox {Q}$ were false and, according to the normic theory, E provides justification for believing $\hbox {P} \,\wedge \, \hbox {Q}$. The explanation for the validity of amalgamation is similar: Suppose E provides justification for believing P and F provides justification for believing P. According to the normic theory, there would have to be some special explanation if E were true and P were false and there would have to be some special explanation if F were true and P were false. What about $\hbox {E} \,\vee \, \hbox {F}$? If $\hbox {E} \,\vee \, \hbox {F}$ were true then *either* E would be true or F would be true. As such, there would have to be some special explanation if $\hbox {E} \,\vee \, \hbox {F}$ were true and P were false and, according to the normic theory, $\hbox {E} \,\vee \, \hbox {F}$ provides justification for believing P.

In thinking about how the normic theory handles other logical principles, however, it may be helpful to approach the theory in a more formal way. Abnormality is not an all-or-nothing notion—it’s plausible that propositions can be placed in some kind of ordering, reflecting how abnormal their truth would be, given background evidence^13^. The maximally normal propositions come first in the ordering and might be assigned a degree of abnormality 0, the next most normal propositions will be assigned a degree of abnormality 1 and so on. On the present conception of normalcy, we might think of the degree of abnormality of a proposition as the *number* of explanations that its truth would require^14^. Suppose we now turn this around—instead of ordering propositions according to how abnormal their truth would be, we order them according to how abnormal their *falsity* would be. In this ordering, the higher the degree of a proposition, the more abnormal its falsity would be, given background evidence—if a proposition has degree 0, then its falsity would not be abnormal at all, if it has degree 12 then its falsity would be highly abnormal etc. Call this the degree to which a proposition is *normically supported* by background evidence. Any body of evidence can now be associated with a normic support function, assigning degrees of normic support to propositions. Like a probability function, a normic support function will assign numbers to propositions in a way that meets certain constraints. But what are these constraints?

The falsity of a contradiction or logical falsehood is never abnormal—as such, the degree of normic support of a logical falsehood will always be 0. The falsity of a logical truth, on the other hand, might be regarded as having infinite abnormality, in which case any logical truth will have an infinite degree of normic support. In order for a conjunction to be false it is enough that one of its conjuncts be false. We might suppose that the falsity of a conjunction $\hbox {P} \,\wedge \, \hbox {Q}$ will be as abnormal as the falsity of P or the falsity of Q, whichever is more normal. We can, at least, adopt this constraint as a working hypothesis—and it would appear to be borne out by the present way of measuring abnormality. In order to explain the falsity of a conjunction it suffices to explain the falsity of either conjunct. It follows that the number of explanations that would be required to explain the falsity of $\hbox {P} \,\wedge \, \hbox {Q}$ will be the number of explanations required to explain the falsity of P or the number of explanations required to explain the falsity of Q, whichever is lower. Given this constraint, the degree to which a conjunction $\hbox {P} \,\wedge \, \hbox {Q}$ is normically supported will be equal to the minimum of the degree to which P is normically supported and the degree to which Q is normically supported.

Suppose again that we have a set of propositions F that is closed under negation and disjunction (conjunction) and includes a maximal proposition entailed by all the others in the set. As before, propositions can be modelled as subsets of a set of possible worlds W, with W serving as the maximal proposition. Let r be a function assigning to each proposition in F the degree of normic support imposed by background evidence. The foregoing reflections give us the following:

1. $$\hbox {r}(\hbox {W}) = \infty $$
2. $$\hbox {r}(\emptyset ) = 0$$
3. $$\hbox {r}(\hbox {P} \,\wedge \, \hbox {Q}) = \hbox {min}(\hbox {r}(\hbox {P}), \hbox {r}(\hbox {Q}))$$

These are nothing other than the axioms for a *positive ranking function* (Spohn 2009, section 2.1, 2012, section 5.3)^15^. Two simple consequences of these principles are worth noting:

(R4) If P entails Q then r(Q) $\ge \hbox {r}(\hbox {P})$
(R5) If $$\hbox {r}(\hbox {P}) > 0$$ then $$\hbox {r}({\sim }\hbox {P}) = 0$$

The degree to which a new piece of evidence E normically supports a proposition P will be equal to the degree of abnormality of $\hbox {E} \,\wedge \,{\sim }\hbox {P}$ minus the degree of abnormality of E. That is, it will be equal to the *extra* abnormality that the falsity of P adds to the existing abnormality of E. Remember, though, that the value assigned to a proposition by r represents how abnormal its *falsity* would be. As such, the degree to which E normically supports P will be equal to $\hbox {r}({\sim }(\hbox {E}~\,\wedge \,{\sim }\hbox {P})) - \hbox {r}({\sim }\hbox {E}) = \hbox {r}({\sim }\hbox {E}~\,\vee \, \hbox {P}) -\hbox {r}({\sim }\hbox {E})$ (where $\infty -\infty = 0$). This matches the standard definition of a *conditional rank* – $\hbox {r}(\hbox {P} \,|\, \hbox {E})$ (see Spohn 2009, section 2.1, 2012, section 5.3). Given this definition, it follows that (R3), (R4) and (R5) also have conditional versions:

(R6) $$\hbox {r}(\hbox {P} \,\wedge \, \hbox {Q} \,\,|\, \hbox {E}) = \hbox {min}(\hbox {r}(\hbox {P} \,|\, \hbox {E}), \hbox {r}(\hbox {Q} \,\,|\, \hbox {E}))$$
(R7) If P entails Q then $\hbox {r}(\hbox {Q} \,\,|\, \hbox {E}) \ge \hbox {r}(\hbox {P} \,|\, \hbox {E})$
(R8) If $$\hbox {r}(\hbox {P} \,|\, \hbox {E}) > 0$$ then $$\hbox {r}({\sim }\hbox {P}~| \hbox {E}) = 0$$.

On the normic theory of justification, $\hbox {E} => \hbox {P iff E}$ provides some positive normic support for P—that is, there will be a normic support function r such that $\hbox {E} =>\hbox { P iff r}(\hbox {P} \,|\, \hbox {E}) > 0$. We might also consider views on which justification corresponds to some higher threshold on this scale – views on which $\hbox {E} => \hbox {P iff r}(\hbox {P} \,|\, \hbox {E}) > \hbox {t}$ for some positive integer t. In (Smith 2016, section 5.2), I referred to these as ‘threshold normic theories’. Interestingly, setting the threshold for justification higher than 0 will make a certain difference to the logical behaviour of $=>$. I’ll note one example of this below, but will otherwise focus on the simple normic theory in which $\hbox {E} => \hbox {P iff r}(\hbox {P} \,|\, \hbox {E}) > 0$.

Return to the Christmas party example and, once again, let T be the proposition that Jen, Bruce and Maude have each testified that they will attend, J be the proposition that Jen will attend, B be the proposition that Bruce will attend and M be the proposition that Maude will attend. On the most natural way of implementing the formalism just developed, we should set $\hbox {r}(\hbox {J}~| \hbox {T}) = \hbox {r}(\hbox {B} \,\,|\, \hbox {T}) = \hbox {r}(\hbox {M} \,\,|\, \hbox {T}) = 1$ where r is my background normic support function, prior to the receipt of the testimony. What this means is that if Jen fails to attend, given the testimony, then a single explanation is needed – and the same goes for Bruce and for Maude. If J, B and M are taken to be mutually independent, it might also make sense to set $\hbox {r}(\hbox {J} \,\vee \, \hbox {B} \,|\, \hbox {T}) = \hbox {r}(\hbox {J} \,\vee \, \hbox {M} \,\,|\, \hbox {T}) = \hbox {r}(\hbox {B} \,\vee \, \hbox {M} \,\,|\, \hbox {T}) = 2$ and $\hbox {r}(\hbox {J} \,\vee \, \hbox {B} \,\vee \, \hbox {M}\,| \hbox {T}) = 3$. In this case, if any two fail to attend then two explanations are needed and if all three fail to attend, three explanations are needed— but these assignments won’t matter for the points I wish to make^16^.

Given that $\hbox {r}(\hbox {J}~| \hbox {T}) = \hbox {r}(\hbox {B} \,|\, \hbox {T}) = \hbox {r}(\hbox {M} \,\,|\, \hbox {T}) = 1$ it follows from (R6) that $\hbox {r}(\hbox {J}~\,\wedge \, \hbox {B} \,|\, \hbox {T}) = \hbox {r}(\hbox {B}~\,\wedge \, ~\hbox {M}~\,|~\hbox {T}) = \hbox {r}(\hbox {J}~\,\wedge \, ~\hbox {M} \,\,|\, \hbox {T}) = \hbox {r}(\hbox {J}\,\wedge \, \hbox {B}~\,\wedge \, \hbox {M} \,|\, \hbox {T}) = 1$. From this, we can prove that $\hbox {r}(\hbox {J}~|~\hbox {T}~\,\wedge \, ~({\sim }\hbox {J} \,\vee \, {\sim }\hbox {B})) = 0$.

### Proof

By the definition of a conditional rank $\hbox {r}(\hbox {J} \,|\, \hbox {T} \,\wedge \, ({\sim }\hbox {J} \,\vee \,{\sim }\hbox {B}))$ is equal to $\hbox {r}({\sim }\hbox {T} \,\vee \, (\hbox {J} \,\wedge \, \hbox {B})\,\vee \, \hbox {J})-\hbox {r}({\sim }\hbox {T}~\,\vee \, (\hbox {J}~\,\wedge \, \hbox {B}))$. By propositional logic, this is equal to $\hbox {r}({\sim }\hbox {T} \,\vee \, \hbox {J}) - \hbox {r}({\sim }\hbox {T}~\,\vee \, (\hbox {J}~\,\wedge \, \hbox {B}))$. This, in turn, is equal to $(\hbox {r}({\sim }\hbox {T}\,\vee \, \hbox {J}) - \hbox {r}({\sim }\hbox {T})) - (\hbox {r}({\sim }\hbox {T}~\,\vee \, ~(\hbox {J}~\,\wedge \, \hbox {B})) - \hbox {r}({\sim }\hbox {T}))$ which, given the definition of a conditional rank, is equal to $\hbox {r}(\hbox {J}\, | \hbox {T}) - \hbox {r}(\hbox {J} \,\wedge \, \hbox {B} \,|\, \hbox {T})$. Given our stipulations, this is equal to 1 – 1 = 0. $\square$

We have it that $\hbox {T}~=>\hbox {J}$ and $(\hbox {T} \,\wedge \, ({\sim }\hbox {J} \,\vee \,{\sim }\hbox {B})) \ne > \hbox {J}$. While I have justification for believing that Jen will attend, this justification is vulnerable to defeat by the proposition that either Jen or Bruce will fail to attend. Monotonicity fails on the normic theory. Further, notice that, by the definition of conditional ranks, $\hbox {r}(\hbox {T}~| \hbox {T} \,\wedge \, ({\sim }\hbox {J}\,\vee \, {\sim }\hbox {B})) = \hbox {r}(\hbox {T} \,\vee \, {\sim }\hbox {T} \,\vee \, (\hbox {J} \,\wedge \, \hbox {B})) - \hbox {r}({\sim }\hbox {T} \,\vee \, (\hbox {J} \,\wedge \, \hbox {B})) = \infty - \hbox {r}({\sim }\hbox {T}~\,\vee \, ~(\hbox {J}~\,\wedge \, ~\hbox {B})) = \infty$. We also have it that $(\hbox {T} \,\wedge \, ({\sim }\hbox {J}\,\vee \, {\sim }\hbox {B})) => \hbox {T}$. $\hbox {T} \,\wedge \, ({\sim }\hbox {J} \,\vee \, {\sim }\hbox {B})$ is the proposition that Jen, Bruce and Maude all said that they will attend, but either Jen or Bruce won’t. This evidence provides justification for believing that Jen, Bruce and Maude all said that they will attend. The evidence that Jen, Bruce and Maude all said that they will attend will, in turn, provide justification for believing that Jen will attend. But the evidence that Jen, Bruce and Maude all said that they will attend, but either Jen or Bruce won’t, does not provide justification for believing that Jen will attend. Simple transitivity fails on the normic theory.

Cumulative transitivity is valid on the normic theory of justification.

### Proof

Suppose $\hbox {E} => \hbox {P}$ and $(\hbox {E} \,\wedge \, \hbox {P}) => \hbox {Q}$. Given the normic theory we have it that $\hbox {r}(\hbox {P} \,|\, \hbox {E}) = \hbox {r}(\hbox {P}~\,\vee \, ~{\sim }\hbox {E}) - \hbox {r}({\sim }\hbox {E}) > 0$ and $\hbox {r}(\hbox {Q}~|~\hbox {E}~\,\wedge \, ~\hbox {P}) = \hbox {r}(\hbox {Q} \,\vee \, {\sim }\hbox {P} \,\vee \,{\sim }\hbox {E}) - \hbox {r}({\sim }\hbox {P} \,\vee \, {\sim }\hbox {E}) > 0$. It follows by (R8) that $\hbox {r}({\sim }\hbox {P} \,|\, \hbox {E}) = \hbox {r}({\sim }\hbox {P}~\,\vee \, ~{\sim }\hbox {E}) - \hbox {r}({\sim }\hbox {E}) = 0$ in which case $\hbox {r}({\sim }\hbox {P}~\,\vee \, ~{\sim }\hbox {E}) = \hbox {r}({\sim }\hbox {E})$. It then follows that $\hbox {r}(\hbox {Q}~\,\vee \, ~{\sim }\hbox {P}~\,\vee \, ~{\sim }\hbox {E}) - \hbox {r}({\sim }\hbox {P} \,\vee \,{\sim }\hbox {E}) = \hbox {r}(\hbox {Q}~\,\vee \, ~{\sim }\hbox {P}~\,\vee \, {\sim }\hbox {E}) - \hbox {r}({\sim }\hbox {E}) > 0$ which is just to say that $\hbox {r}(\hbox {Q}~\,\vee \, ~{\sim }\hbox {P}~|~\hbox {E}) > 0$. Since $\hbox {P} \,\wedge \, (\hbox {Q} \,\vee \,{\sim }\hbox {P})$ is equivalent to $\hbox {P}~\,\wedge \, ~\hbox {Q}$, if we have $\hbox {r}(\hbox {P}~|~\hbox {E}) > 0$ and we have $\hbox {r}(\hbox {Q} \,\vee \,{\sim }\hbox {P} \,|\, \hbox {E}) > 0$ it follows, by (R6), that $\hbox {r}(\hbox {P}~\,\wedge \, ~\hbox {Q} \,\,|\, \hbox {E}) > 0$. By (R7) it follows that $\hbox {r}(\hbox {Q} \,\,|\, \hbox {E}) > 0$ and $\hbox {E} => \hbox {Q}$ as required. $\square$

Cautious monotonicity is valid on the normic theory of justification.

### Proof

Suppose $\hbox {E} => \hbox {P}$ and $\hbox {E} => \hbox {Q}$. Given the normic theory we have it that $\hbox {r}(\hbox {P} \,|\, \hbox {E}) = \hbox {r}(\hbox {P}~\,\vee \,\, {\sim }\hbox {E}) - \hbox {r}({\sim }\hbox {E}) > 0$ and $\hbox {r}(\hbox {Q}~\,|~\hbox {E}) = \hbox {r}(\hbox {Q} \,\vee \,\, {\sim }\hbox {E}) - \hbox {r}({\sim }\hbox {E}) > 0$. It follows by (R8) that $\hbox {r}({\sim }\hbox {Q} \,|\, \hbox {E}) = \hbox {r}({\sim }\hbox {Q}~\,\vee \, ~{\sim }\hbox {E}) - \hbox {r}({\sim }\hbox {E}) = 0$ in which case $\hbox {r}({\sim }\hbox {Q}~\,\vee \, ~{\sim }\hbox {E}) = \hbox {r}({\sim }\hbox {E})$. It then follows that $\hbox {r}(\hbox {P} \,\vee \, {\sim }\hbox {E}) - \hbox {r}({\sim }\hbox {Q} \,\vee \,{\sim }\hbox {E}) > 0$ and since, by (R4), $\hbox {r}(\hbox {P} \,\vee \,{\sim }\hbox {Q} \,\vee \, {\sim }\hbox {E}) \ge \hbox {r}(\hbox {P} \,\vee \, {\sim }\hbox {E})$ we have it that $\hbox {r}(\hbox {P} \,\vee \,{\sim }\hbox {Q} \,\vee \, {\sim }\hbox {E}) - \hbox {r}({\sim }\hbox {Q}\,\vee \,{\sim }\hbox {E}) > 0$, which is just to say that $\hbox {r}(\hbox {P} \,|\, \hbox {E} \,\wedge \, \hbox {Q}) > 0$ and $\hbox {E} \,\wedge \, \hbox {Q} => \hbox {P}$ as required. $\square$

Since this proof only uses $\hbox {r}(\hbox {Q}\,~|~\hbox {E}) > 0$ to derive the weaker $\hbox {r}({\sim }\hbox {Q} \,|\, \hbox {E}) = 0$, which also follows from $\hbox {E}~\ne >~{\sim }\hbox {Q}$ we can see that rational monotonicity is also valid on the normic theory of justification^17^.

In this paper, I described a method for assessing theories of justification—an alternative, or a supplement, to the way in which such theories are standardly assessed. Rather than looking at what a theory of justification predicts about hypothetical cases, I’ve suggested that we look at what it predicts about putative *logical principles* for justification. I also attempted, in a preliminary way, to put this method into practice. I argued in favour of five logical principles for justification—conjunction closure, cumulative transitivity, cautious and rational monotonicity and amalgamation—and argued against two—simple transitivity and monotonicity. I showed that, while all these principles are violated on the risk minimisation theory, and all these principles are validated on the infallibilist theory, the normic theory strikes the right balance. While I take this to be a significant advantage for the normic theory, my aim here is not to draw a final verdict about this, or any other, approach.

## Footnotes

1 A normal modal logic is one that includes all instances of the **K** schema and the **Necessitation** and **Modus Ponens** rules. The validity of this inference pattern can easily be proved using these resources.

2 The following ‘Ascent’ and ‘Descent’ principles for justification have been the focus of some recent, much deserved, attention (see for instance, Smithies 2012, Rosenkranz forthcoming):

$$\begin{aligned}&\underline{\hbox {JP }}\qquad \underline{{\sim }\hbox {JP }}\qquad \underline{\hbox {JJP}} \qquad \underline{\hbox {J}{\sim }\hbox {JP}}\\&\hbox {JJP} \qquad \hbox {J}{\sim }\hbox {JP} \qquad \hbox {JP} \qquad {\sim }\hbox {JP} \end{aligned}$$

I don’t consider these principles in the present paper.

3 What I’m proposing is not by any means a ‘new’ method—I’m merely importing, into debates over the nature of epistemic justification, a method that is very familiar in other areas. The method is commonplace even in areas that are ostensibly related to (though seldom appealed to in) debates over epistemic justification—such as the literature on nonmonotonic logic or the literature on the logic of confirmation stemming in part from the work of Hempel (1945). I will have a little more to say about these areas along the way.

4 From the claim that justification requires evidential certainty, it won’t immediately follow that justification is beyond reach—we also need the supposition that evidential certainty is beyond reach. The idea that the vast majority of our beliefs are underdetermined by the evidence we possess is one that is very widespread—and perhaps even a part of common sense—but it can be avoided if we adopt a generous account of when a proposition can count as a part of one’s evidence (see for instance Klein 1995, Williamson 2000, chaps. 9, 10). Whatever we make of this strategy, there are other, more subtle, criticisms of the infallibilist theory that cannot be avoided in this way. I will note one such criticism in Sect. 4.

5 Douven and Williamson (2006, section 1) consider a related (stronger) principle: If evidence E provides justification for believing each of a set of propositions $\hbox {P}_{1}$, $\hbox {P}_{2}$...$\hbox {P}_{\mathrm{n}}$ and $\hbox {E} \,\wedge \, \hbox {P}_{1} \,\wedge \, \hbox {P}_{2} \,\wedge \,{\ldots }\,\wedge \, \hbox {P}_{\mathrm{n}}$ provides justification for believing Q then E provides justification for believing Q. Douven and Williamson deploy this principle in order to argue for conjunction closure, which can be derived from it (given some very weak assumptions). In my view, the fact that this principle entails conjunction closure (and does so fairly obviously) makes it *less* dialectically effective for use against the risk minimisation theory—it’s difficult to see why the failure of this principle should present any *additional* cost for the theory over and above the costs of abandoning conjunction closure. The principle in the body text, in contrast, does not commit one to conjunction closure.

6 The ascent and descent principles outlined in n2 can also be written in the new notation where they will feature embedded occurrences of the $=>$ operator and, like conjunction closure, will exhibit no variation in the antecedent term.

7 While it can be helpful to think of cumulative transitivity as a weakening of simple transitivity, the former cannot, in fact, be derived from the latter alone, though certain very weak principles will suffice for the derivation (one example is the principle that Hawthorne (1996, pp. 196) calls ‘Weak And’.

8 It is well known that the principle of deductive closure can be employed in both sceptical arguments and in neo-Moorean responses to scepticism, since many of our ordinary beliefs entail the negations of sceptical hypotheses. Suppose again that I wander into a room I’ve never been in before and notice that the wall before me appears to be red. The proposition that the wall is red entails that the wall is not white and illuminated by tricky red light. As such, if we assume deductive closure, and take it as a starting point that I lack justification for believing that the wall is not white and illuminated by tricky red light, we can derive the result that I lack justification for believing that the wall is red. By the same token, if we assume deductive closure, and take it as a starting point that I have justification for believing that the wall is red, we can derive the result that I have justification for believing that the wall is not white and illuminated by tricky red light. This is familiar—but what is less familiar is that the principle of rational monotonicity can play a similar role, and can actually be used to drive a *broader range* of sceptical and neo-Moorean arguments. Instead of the hypothesis that the wall is white and illuminated by tricky red light, consider the simpler hypothesis that the wall is illuminated by tricky red light. The proposition that the wall is red does not entail that it is not illuminated by tricky red light—so the principle of deductive closure places no restrictions on how the justificatory status of these two propositions are related. But if I were to discover that the wall is illuminated by tricky red light then, although this is consistent with the wall being red, it would clearly *defeat* my justification for believing that it is (a standard example of a so-called ‘undercutting’ defeater). As such, if we assume rational monotonicity, and take it as a starting point that I lack justification for believing that the wall is not illuminated by tricky red light, we can derive the result that I lack justification for believing that the wall is red. By the same token, if we assume rational monotonicity, and take it as a starting point that I have justification for believing that the wall is red, we can derive the result that I have justification for believing that the wall is not illuminated by tricky red light. Thanks here to an anonymous referee for prompting me to think further about connections between rational monotonicity, scepticism and neo-Mooreanism.

9 In n3 I suggested that debates over the nature of epistemic justification and debates over the logic of confirmation bore a close connection. The risk minimisation conception has an analogue in those debates—namely the ‘high probability criterion of confirmation’, according to which, evidence E confirms proposition P just in case $\hbox {Pr}(\hbox {P} \,|\, \hbox {E}) > \hbox {t}$ where t is a value close to, but less than, one (see, for instance, Niinuluoto 2007, section 4.2). The two views will be equivalent on the assumption that evidence E confirms proposition P just in case E provides justification for believing P.

10 While it is standard to define conditional probability in terms of unconditional probability, there are alternative axiomatisations that treat conditional probability as the primitive notion, such as the following:

1. There are propositions P and E such that $\hbox {Pr}(\hbox {P} \,|\, \hbox {E}) < 1$
2. If E entails P then $\hbox {Pr}(\hbox {P} \,|\, \hbox {E}) = 1$
3. If P and Q are inconsistent, given E, then either $\hbox {Pr}(\hbox {P} \,\vee \, \hbox {Q} \,\,|\, \hbox {E}) = \hbox {Pr}(\hbox {P} \,|\, \hbox {E}) + \hbox {Pr}(\hbox {Q} \,\,|\, \hbox {E}) \hbox { or Pr}(\hbox {X}~\,|~\hbox {E}) = 1$ for every proposition X.
4. $$\hbox {Pr}(\hbox {P} \,\wedge \, \hbox {Q} \,\,|\, \hbox {E}) = \hbox {Pr}(\hbox {P} \,|\, \hbox {Q} \,\wedge \, \hbox {E}).\hbox {Pr}(\hbox {Q} \,\,|\, \hbox {E})$$

A *conditional probability function* Pr takes each *pair* of propositions in F to a number in a way that conforms to the above axioms. This is, in essence, the axiomatisation described by Hawthorne (2007, section 2)—though Hawthorne, taking probability functions to be defined over the sentences of a formal language, rather than propositions modelled as sets, requires an additional axiom to ensure that conditional probability functions do not discriminate between logically equivalent sentences. Hawthorne’s axiomatisation of conditional probability is equivalent to that famously described by Popper (Popper 1986, new appendix iv, see Hawthorne 1996, section 2). The unconditional probability of a proposition can now be defined as the probability of that proposition conditional upon W—a definition that will allow us to recapture the classical axioms as theorems, as well as the ratio formula. That is, given this definition of unconditional probability, we can prove that $\hbox {Pr}(\hbox {P}~|~\hbox {E}) = \hbox {Pr}(\hbox {P}~\,\wedge \, ~\hbox {E})/\hbox {Pr}(\hbox {E})$ whenever $\hbox {Pr}(\hbox {E}) > 0$. This approach may have some advantages, particularly if our objective is to use a conditional probability threshold to supply a truth condition for a conditional operator $=>$. (CP2) guarantees that, for any threshold value, $\hbox {E} => \hbox {P}$ will hold whenever E entails P—that is, $\hbox {E} => \hbox {P}$ will be a theorem whenever $\hbox {E}\supset \hbox {P}$ is a theorem (in accordance with the **Deduction with Conditionals** or **RCK** rule for conditional logic). On the classical approach, $=>$ will lack this formal feature (and will thus fail to count as a ‘normal’ conditional operator), as $\hbox {Pr}(\hbox {P} \,|\, \hbox {E})$ may still be undefined when E entails P. The ‘Pr’s in the body text can, in any case, be interpreted as either classical or conditional probability functions. Indeed, the differences between these two approaches will not assume any significance for present purposes, with one notable exception which I will discuss in n12.

11 Both simple and cumulative transitivity are special cases of the following pattern, which we might call *full* or *generalised* transitivity:

$$\begin{aligned}&(\hbox {F} \,\wedge \,\hbox {E}) => \hbox {P}\\&\underline{(\hbox {F} \,\wedge \, \hbox {P}) => \hbox {Q}}\\&(\hbox {F} \,\wedge \, \hbox {E}) => \hbox {Q} \end{aligned}$$

If we let F = W this collapses into simple transitivity, while if we let F = E this collapses into cumulative transitivity. Generalised transitivity is also valid on the infallibilist theory.

12 If Pr were interpreted as a *conditional probability* function, rather than a classical function, this may lead to a certain improvement in the formal scorecard for infallibilism. The most we could then prove, regarding monotonicity, is that $\hbox {Pr}(\hbox {P}~|~\hbox {E}~\,\wedge \, ~\hbox {Q}) = 1$ will follow from $\hbox {Pr}(\hbox {P} \,|\, \hbox {E}) = 1$ if $\hbox {Pr}(\hbox {Q} \,\,|\, \hbox {E}) > 0$ which is just to say that $\hbox {Pr}(\hbox {P}~|~\hbox {E}) = 1$ and $\hbox {Pr}({\sim }\hbox {Q} \,\,|\, \hbox {E}) < 1$ entails $\hbox {Pr}(\hbox {P}~|~\hbox {E}~\,\wedge \, ~\hbox {Q}) = 1$—which is, in effect, *rational* monotonicity. Furthermore, if Pr is a conditional probability function and $\hbox {Pr}(\hbox {E}) = \hbox {Pr}(\hbox {E} \,|\, \hbox {W}) = 0$, then $\hbox {Pr}(\hbox {P} \,|\, \hbox {E})$, $\hbox {Pr}(\hbox {Q} \,\,|\, \hbox {P})$ and $\hbox {Pr}(\hbox {Q}~|~\hbox {E})$ may still be defined and, unconstrained by the ratio formula, may take values that violate simple transitivity. It will still be possible of course to prove that $\hbox {Pr}(\hbox {Q}~|~\hbox {E}) = 1$ will follow from $\hbox {Pr}(\hbox {P} \,|\, \hbox {E}) = 1$ and $\hbox {Pr}(\hbox {Q}~|~\hbox {P}) = 1$ provided $\hbox {Pr}(\hbox {E}) > 0$.

13 It feels somewhat artificial to speak about the abnormality of *propositions*. As alluded to earlier, the terms ‘normal’ and ‘abnormal’ are more often applied to events or states of affairs—though an assessment of the abnormality of an event or state of affairs can always be paraphrased as an assessment of the abnormality of the truth of a proposition. The situation may not be altogether different with terms such as ‘likely’, ‘unlikely’, ‘probable’ and ‘improbable’ which are also often applied to events or states of affairs.

14 This is merely employed here as a rough heuristic for helping us to think through possible logical constraints on degrees of abnormality. It is not intended as an ‘operational’ definition that would allow us to determine the degree of abnormality of any proposition. In many cases, it may be unclear how to ‘count up’ the number of explanations that a proposition would require, owing to difficulties in the identification and individuation of explanations. I won’t discuss this further here.

15 Spohn also uses ranking theory to explicate the logic of normal and abnormal conditions (see Spohn 2012, sections 13.1, 13.2, 2014). Spohn has a different, though related, aim to mine—namely, to provide an analysis of ceteris paribus laws, and the ways in which they tolerate exceptions. Spohn also works with a different conception of normalcy to the one I have outlined here—indeed a conception which is epistemic from the start, as opposed to a non-epistemic conception which the normic theory imbues with a certain epistemic significance. I won’t pursue this comparison further here. The notion of normalcy appealed to here also has its origins in a certain treatment of ceteris paribus laws—namely, that provided by Pietroski and Rey (1995), (see also Smith 2007, 2016, sections 2.3, 6.2).

16 Relative to a ranking function r two proposition X and Y are said to be independent, given Z, just in case $\hbox {r}(\hbox {X}~\,\vee \, ~\hbox {Y}\, \,|\, \hbox {Z}) + \hbox {r}({\sim }\hbox {X}~\,\vee \, ~{\sim }\hbox {Y} \,\,|\, \hbox {Z}) = \hbox {r}({\sim }\hbox {X}~\,\vee \, ~\hbox {Y} \,\,|\, \hbox {Z}) + \hbox {r}(\hbox {X}~\,\vee \, ~{\sim }\hbox {Y} \,\,|\, \hbox {Z})$ (see Spohn 2012, section 7.1) If, relative to r, J, B and M are mutually independent given T then, given a few further natural stipulations, we arrive at the assignments in the text. There may be reasons however to question these assignments, and thus question whether J, B and M should be regarded as mutually independent in this sense. It may be that there are possible factors (a storm, a road closure) which could *simultaneously* explain the failure of Jen, Bruce and Maude to attend. In this case, the falsity of $\hbox {J} \,\vee \, \hbox {B} \,\vee \, \hbox {M}$ given T *may* have multiple explanations but would only *require* a single explanation and we may wish to set $\hbox {r}(\hbox {J} \,\vee \, \hbox {B}\, | \hbox {T}) = \hbox {r}(\hbox {J}~\,\vee \, ~\hbox {M} \,|\, \hbox {T}) = \hbox {r}(\hbox {B} \,\vee \, \hbox {M} \,\,|\, \hbox {T}) = \hbox {r}(\hbox {J}~\,\vee \, \hbox {B} \,\vee \, \hbox {M} \,\,|\, \hbox {T}) = 1$. This is still consistent with the propositions being mutually *probabilistically* independent and, as mentioned, won’t affect any of the points made in the main text. Thanks here to an anonymous referee.

17 If the threshold for justification t were set higher than 0 then $\hbox {r}({\sim }\hbox {Q} \,\,|\, \hbox {E}) = 0$ would no longer follow from $\hbox {E}\ne >~{\sim }\hbox {Q}$—all that would follow is $\hbox {r}({\sim }\hbox {Q} \,\,|\, \hbox {E}) \le \hbox {t}$. But $\hbox {r}({\sim }\hbox {Q} \,\,|\, \hbox {E}) = \hbox {r}({\sim }\hbox {Q}~\,\vee \, ~{\sim }\hbox {E}) - \hbox {r}({\sim }\hbox {E}) \le \hbox {t}$ and $\hbox {r}(\hbox {P} \,|\, \hbox {E}) = \hbox {r}(\hbox {P}~\,\vee \, {\sim }\hbox {E}) - \hbox {r}({\sim }\hbox {E}) > \hbox {t}$ do not entail that $\hbox {r}(\hbox {P} \,\vee \,{\sim }\hbox {E}) - \hbox {r}({\sim }\hbox {Q} \,\vee \, {\sim }\hbox {E}) > \hbox {t}$. As such, the above reasoning would no longer go through as a proof of rational monotonicity (though it would still work as a proof of cautious monotonicity since $\hbox {r}({\sim }\hbox {Q}~|~\hbox {E}) = 0$ still follows from $\hbox {r}(\hbox {Q} \,\,|\, \hbox {E}) > \hbox {t})$. In fact, it can be shown that rational monotonicity is not valid on a ‘threshold’ normic theory of justification.

## References

[CR1] Bergmann M (2006). Justification Without Awareness: A Defense of Epistemic Externalism.

[CR2] Conee E, Feldman R, Smith Q (2008). Evidence. Epistemology: New Essays.

[CR3] Dougherty T, Dougherty T (2011). In defence of propositionalism about evidence. Evidentialism and its Discontents.

[CR4] Douven I, Williamson T (2006). Generalizing the lottery paradox. British Journal for the Philosophy of Science.

[CR5] Fantl J, McGrath M (2011). Knowledge in an Uncertain World.

[CR6] Foley R (1993). Working Without a Net.

[CR7] Foley R, Huber F, Schmidt-Petri C (2009). Beliefs, degrees of belief and the Lockean thesis. Degrees of Belief.

[CR8] Hawthorne J (1996). On the logic of nonmonotonic conditionals and conditional probabilities. Journal of Philosophical Logic.

[CR9] Hawthorne J (2007). Nonmonotonic conditionals that behave like conditional probabilities above a threshold. Journal of Applied Logic.

[CR10] Hempel, C. (1945). Studies in the logic of confirmation. *Mind*, *v54*, 1–26 and 97–121.

[CR11] Klein P (1995). Scepticism and closure: Why the evil genius argument fails. Philosophical Topics.

[CR12] Kyburg H, Swain M (1970). Conjunctivitis. Induction, Acceptance and Rational Belief.

[CR13] Lehman D, Magidor M (1992). What does a conditional knowledge base entail?. Artificial Intelligence.

[CR14] Niinuluoto I, Kuipers T (2007). Evaluation of theories. General Philosophy of Science, Focal Issues.

[CR15] Pietroski P, Rey G (1995). When other things aren’t equal: Saving “ceteris paribus” laws from vacuity. British Journal for the Philosophy of Science.

[CR16] Pollock J (1983). Epistemology and probability. Synthese.

[CR17] Popper K (1968). The Logic of Scientific Discovery.

[CR18] Rosenkranz, S. (forthcoming) ‘The structure of justification’ *Mind*

[CR19] Smith M (2007). Ceteris paribus conditionals and comparative normalcy. Journal of Philosophical Logic.

[CR20] Smith M (2010). What else justification could be. Noûs.

[CR21] Smith M (2016). Between Probability and Certainty: What Justifies Belief.

[CR22] Smithies D (2012). Moore’s paradox and the accessibility of justification. Philosophy and Phenomenological Research.

[CR23] Spohn W, Huber F, Schmidt-Petri C (2009). Survey of ranking theory. Degrees of Belief.

[CR24] Spohn W (2012). The Laws of Belief: Ranking Theory and its Philosophical Applications.

[CR25] Spohn W (2014). The epistemic account of ceteris paribus conditions. European Journal for the Philosophy of Science.

[CR26] Williamson T (2000). Knowledge and its Limits.

